# P-1944. The Natural History of SARS-CoV-2 Infection in Infants and the Role of Subsequent Infections on Protection: A Prospective Cohort in Mexico City

**DOI:** 10.1093/ofid/ofae631.2103

**Published:** 2025-01-29

**Authors:** Alejandra Molina-Sanchez, Laura Angelica Rodriguez-Dorantes, Sandra Rajme-López, Pilar Ramos-Cervantes, Allison R Cline, Mary A Staat, Guillermo Miguel Ruiz-Palacios

**Affiliations:** Instituto Nacional de Ciencias Médicas y Nutrición Salvador Zubirán, Mexico City, Distrito Federal, Mexico; Instituto Nacional de Ciencias Médicas y Nutrición Salvador Zubirán, Mexico City, Distrito Federal, Mexico; Instituto Nacional de Ciencias Médicas y Nutrición Salvador Zubirán, Mexico City, Distrito Federal, Mexico; Instituto Nacional de Ciencias Médicas y Nutrición Salvador Zubirán, Mexico City, Distrito Federal, Mexico; University of Cincinnati College of Medicine, Cincinnati, Ohio; Cincinnati Children’s Hospital Medical Center, Cincinnati, Ohio; Instituto Nacional de Ciencias Médicas y Nutrición Salvador Zubirán, Mexico City, Distrito Federal, Mexico

## Abstract

**Background:**

Limited information exists regarding the natural history of SARS-CoV-2 infection in infants, including the proportion of asymptomatic disease. Moreover, the impact of repeated infections on immunity and clinical presentation is unknown. The possibility of non-severe symptoms related to viral variants has not been explored.

Incidence rate of SARS-CoV-2 first and subsequent infections.

**Figure:**
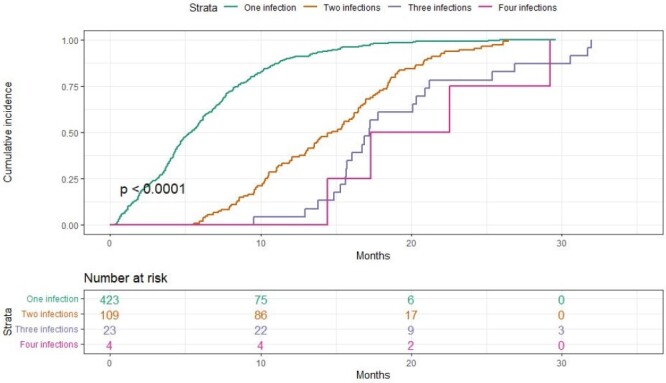

**Methods:**

This meticulous prospective cohort study, conducted from January 1st 2022 to March 30th 2024, followed healthy newborns with weekly rtPCR. Upon respiratory symptoms or a positive rtPCR, subjects were evaluated and a daily symptomatic record was kept for 14 days. Positive samples were sequenced. Incidence rates were calculated for infection and reinfection. Symptoms were grouped according to organ and system involvement. Descriptive and logistic regression analyses were performed in R 4.3.3.

**Figure:**
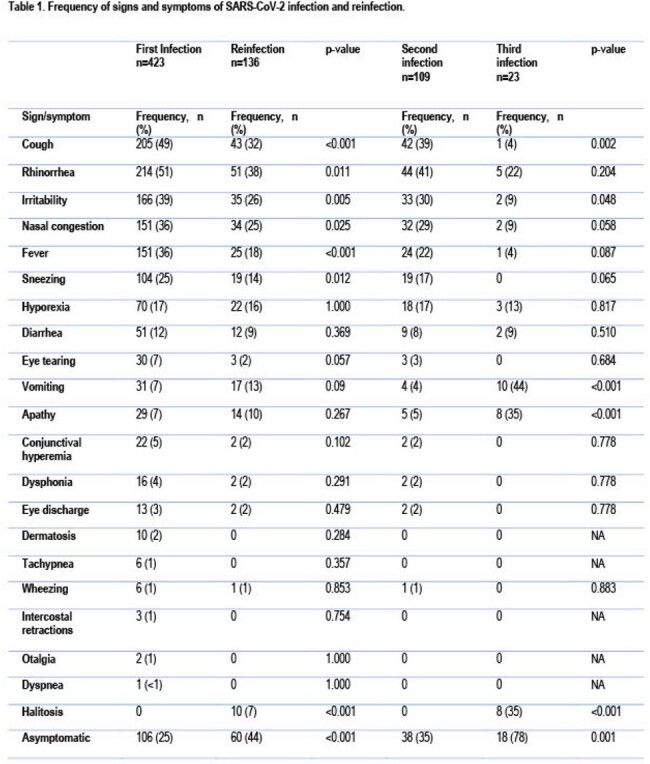

**Results:**

Of 1181 eligible newborns, 796 were included and followed for 10,774 child-months. Nasal swab yield was 76%. Fifty two percent were male, 67% were exclusively breastfed, and none were vaccinated against SARS-CoV-2. A total of 559 SARS-CoV-2 infections were detected (423 first, 109 second, 23 third, and 4 fourth infections). The incidence rate was 5.1 cases per 100 child-months (Figure 1). One subject had severe COVID-19.

The most common symptoms for first infection were rhinorrhea (51%), cough (49%), irritability (39%), and fever (36%). Asymptomatic infection was more common in reinfections than first infections (44 vs 25%, p=0.001) (Table 1). The frequency of asymptomatic infection progressively increased from 25% to 35% and 78% for the first, second, and third infections (p 0.02; X^2^ for trend).

Subjects with throat, eyes, and skin symptoms were more likely to be infected with the BA.1/BA.2 variants. Those with systemic disease were more likely to be infected by the BA.4/BA.5. Asymptomatic infection tended to be more common in infections by the XBB/EG variants (Table 2). The odds of having an asymptomatic infection increased in subjects with ≥ 2 infections. (Table 3).

**Figure:**
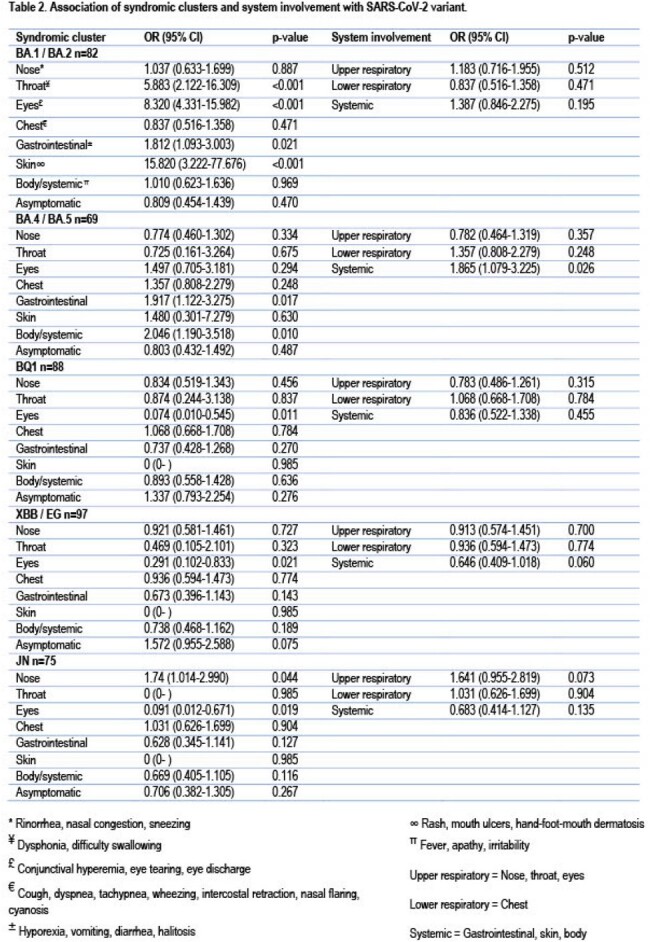

**Conclusion:**

SARS-CoV-2 infection is common in infants, though predominantly non-severe. Asymptomatic infections are frequent. Subsequent infections conferred protection against symptomatic infection. The clinical characteristics of COVID-19 differ according to the viral variant.

**Figure:**
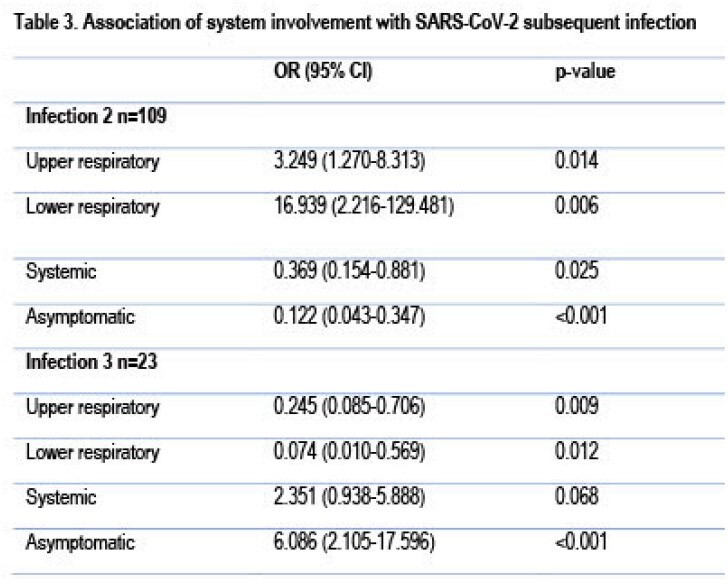

**Disclosures:**

Mary A. Staat, MD, MPH, Cepheid: Grant/Research Support|Merck: Grant/Research Support|Pfizer: Grant/Research Support|Up-To-Date: Honoraria

